# Marked aggravation of pyrethroid resistance in major malaria vectors in Malawi between 2014 and 2021 is partly linked with increased expression of P450 alleles

**DOI:** 10.1186/s12879-022-07596-9

**Published:** 2022-07-30

**Authors:** Benjamin D. Menze, Magellan Tchouakui, Leon M. J. Mugenzi, Williams Tchapga, Micareme Tchoupo, Murielle J. Wondji, Martin Chiumia, Themba Mzilahowa, Charles S. Wondji

**Affiliations:** 1 Medical Entomology Department, Centre for Research in Infectious Diseases (CRID), Yaoundé, Cameroon; 2 Vector Biology Department, Liverpool School of Tropical Medicine, Pembroke Place, Liverpool, L3 5QA UK; 3 Malaria Alert Centre (MAC), Kamuzu University of Health Sciences (KUHeS), Blantyre, Malawi

**Keywords:** *A. funestus*, Super-resistance, Pyrethroids, Malawi, Malaria, Metabolic resistance

## Abstract

**Background:**

Increased intensity of pyrethroid resistance is threatening the effectiveness of insecticide-based interventions to control malaria in Africa. Assessing the extent of this aggravation and its impact on the efficacy of these tools is vital to ensure the continued control of major vectors. Here we took advantage of 2009 and 2014 data from Malawi to establish the extent of the resistance escalation in 2021 and assessed its impact on various bed nets performance.

**Methods:**

Indoor blood-fed and wild female Anopheles (An) mosquitoes were collected with an electric aspirator in Chikwawa. Cocktail and SINE PCR were used to identify sibling species belonging to *An. funestus* group and *An. gambiae* complex. The susceptibility profile to the four classes of insecticides was assessed using the WHO tubes bioassays. Data were saved in an Excel file. Analysis was done using Vassarstats and figures by Graph Pad.

**Results:**

In this study, a high level of resistance was observed with pyrethroids (permethrin, deltamethrin and alpha-cypermethrin with mortality rate at 5x discriminating concentration (DC) < 50% and Mortality rate at 10x DC < 70%). A high level of resistance was also observed to carbamate (bendiocarb) with mortality rate at 5x DC < 25%). Aggravation of resistance was also noticed between 2009 and 2021. For pyrethroids, the mortality rate for permethrin reduced from 47.2% in 2009 to 13% in 2014 and 6.7% in 2021. For deltamethrin, the mortality rate reduced from 42.3% in 2009 to 1.75% in 2014 and 5.2% in 2021. For Bendiocarb, the mortality rate reduced from 60% in 2009 to 30.1% in 2014 and 12.2% in 2021. The high resistance observed is consistent with a drastic loss of pyrethroid-only bed nets efficacy although Piperonyl butoxide (PBO)-based nets remain effective. The resistance pattern observed was linked with high up-regulation of the P450 genes *CYP6P9a, CYP6P9b* and *CYP6M7* in *An. funestus* s.s. mosquitoes surviving exposure to deltamethrin at 1x, 5x and 10x DC. A significant association was observed between the 6.5 kb structural variant and resistance escalation with homozygote resistant (SV+/SV+) more likely to survive exposure to 5x and 10x (OR = 4.1; P < 0.001) deltamethrin than heterozygotes. However, a significant proportion of mosquitoes survived the synergist assays with PBO suggesting that other mechanisms than P450s are present.

**Conclusions:**

This resistance aggravation in *An. funestus* s.s. Malawian population highlights an urgent need to deploy novel control tools not relying on pyrethroids to improve the effectiveness of vector control.

**Supplementary Information:**

The online version contains supplementary material available at 10.1186/s12879-022-07596-9.

## Background

Vector control interventions through long-lasting insecticidal nets (LLINs) and indoor residual spraying (IRS) have led to a decline in malaria incidence of about 80% in the past decade [1]. Despite this meaningful reduction, efforts to mitigate this disease have been slowed in recent years, with evidence of an escalation in malaria cases from 227 to 241 million cases between 2019 and 2020, including 627,000 deaths worldwide [2]. Unfortunately, this escalation in malaria incidence has been strongly linked to the wide-spread of insecticide resistance in the major malaria vectors and mainly in Africa, as Africa alone hosts about 94% of the global malaria burden [3–6]. Indeed, the massive use of agrochemical compounds in agriculture, combined with the scale-up of insecticide-based vector control tools including novel nets like Royal guard and Interceptor G2, has partly contributed to strong selection pressure on the field and especially on the breeding sites, allowing some mosquito populations to adapt and survive exposure to multiple insecticides at the discriminating concentration (1x DC) and even at 5x and/or 10x (5x, 10x) the DC [7, 8].

Although malaria has been reduced in some African countries, the burden still remains high in Malawi, where vector control is principally based on the use of LLINs and IRS so far [6, 9–12]. Evidence of worsening of resistance against the principal class of insecticide (pyrethroid) used to contain the spread of malaria has been reported in southern Malawi using time exposure. This increase of resistance was consistent with the loss of efficacy of bed nets in southern Malawi and Mozambique [11]. Depending on the selection pressure, insecticide resistance could increase over time with use of bed nets as reported in some African Regions in *An. funestus* and *An. gambiae* [8, 13–15]. In most cases, the investigation of resistance patterns in malaria-transmitting vectors has been done using the diagnostic dose (1x) of insecticide recommended by the World Health Organization (WHO) or using the Centres for Disease Control and Prevention (CDC) Bottle assay [16]. This dose of insecticide only indicates whether a mosquito population is resistant or not, but does not give the intensity or degree of resistance in the population that survived the diagnostic dose of exposure. For the past years, some studies have used the time exposure which is not a standardized method to access the strength of resistance in *Anopheles* species [4, 11]. To date, to quantify the strength of resistance in field population, WHO has set up a guideline based on the use of high concentrations of insecticide (5x DC and 10 DC). Based on the previous study done in Malawi to access the increase of resistance intensity to multiple insecticides by using time exposure where it was reported high levels of resistance with evidence of reduced standard bed nets efficacy, we hypothesized that resistance pattern is worsening in southern Malawi in malaria vectors allowing them to become super-resistant to the latest LLINs such as Royal Guard and Interceptor G2 deployed in the field to supplement existing vector control tools. Assessing the resistance intensity on the major malaria vectors as well as the mechanisms by which this occurs is very important to anticipate the spread of such super-resistance regionally or continent-wide and to better plan for the implementation of new intervention strategies tools in the field to achieve the elimination of malaria as set by the WHO.

This study was designed to investigate the resistance intensity profile of *Anopheles* species in southern Malawi (Chikwawa), the molecular drivers implicated and the impact of such super-resistance on the effectiveness of LLINs.

## Methods

### Study site and mosquito collection

**Fig. 1:**
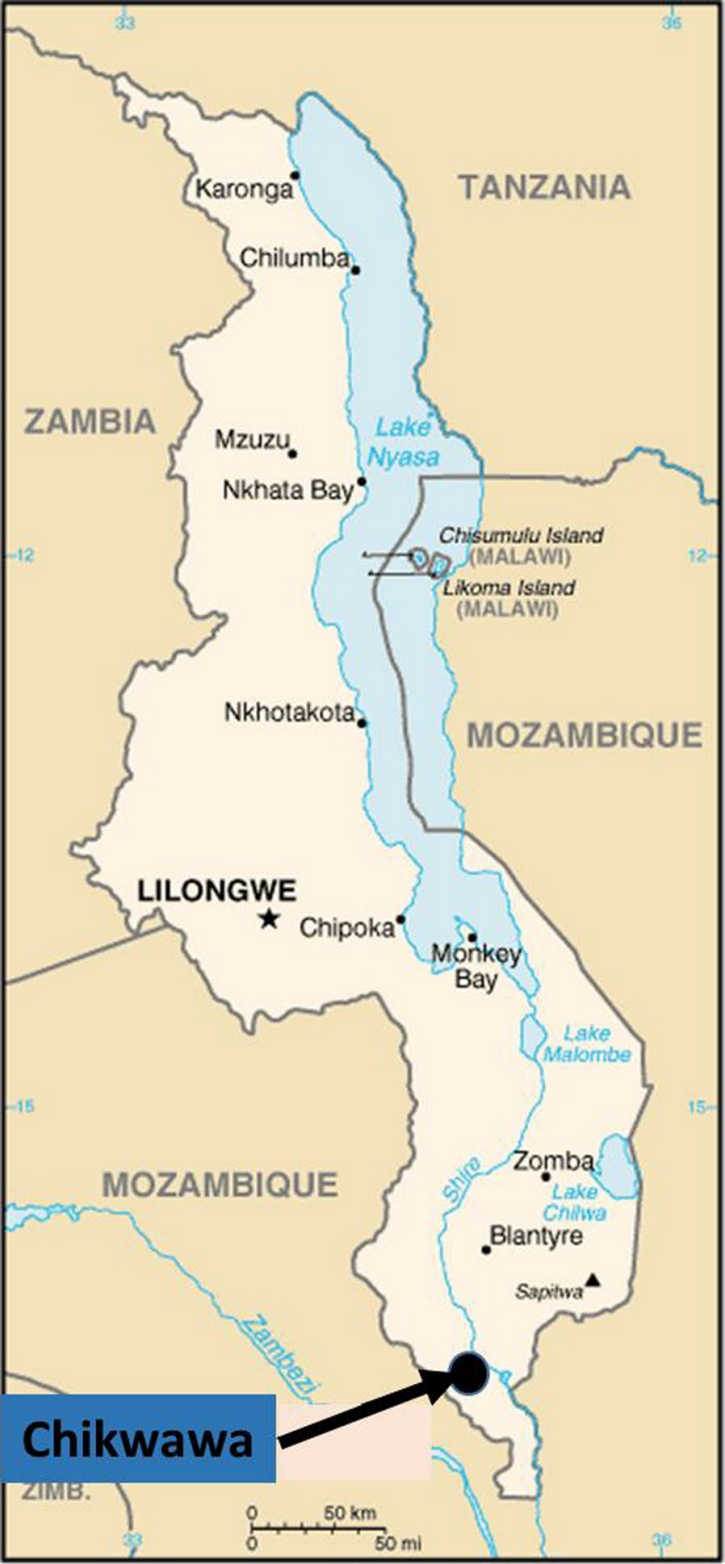
Map of the study site showing the locality of Chikwawa

Indoor blood-fed and wild female *Anopheles* mosquitoes resting on the walls and roofs of houses were collected with an electric aspirator in three randomly selected areas of Chikwawa in southern Malawi [Npangeni (Latitude: S 16° 2′ 8′′, Longitude: E 34° 50′ 21′′, Altitude: 63 m], Medrum I and Medrum II [Latitude: S 16° 2′3′′, Longitude: E 34° 50′ 9′′, Altitude: 74 m)]. Collection was carried out over seven (7) days during June 2021 in at least five (05) randomly selected houses in each village (Fig. 1). Before the sampling of mosquitoes in these villages, a questionnaire was designed to collect information regarding the type of houses, the number of inhabitants, the type of bed net used, and the number of people protected. Verbal consent was obtained from the head of each family and the chief of each village before collection.

All *Anopheles* mosquitoes collected were morphologically identified as belonging to *An. funestus* group or *An. gambiae* complex according to morphological keys [17, 18]. All blood fed mosquitoes were then kept in paper cups and fed with 10% sugar solution for about 4 to 5 days to allow them to be fully gravid. Afterward, they were forced to lay eggs in 1.5 ml Eppendorf tubes following the method previously described [19]. Eggs of the oviposited mosquitoes were reared in the insectarium under standard conditions using mineral water and TetraMin baby fish food till the adult stage for the WHO susceptibility test [16]. A subset of non-oviposited females was kept in Eppendorf tubes containing silica gel for further molecular analyses. The samples from 2009 to 2014 were also collected in Chikwawa area and were mainly member of *An. funestus* group [20, 21].

### Molecular identification of field collected mosquitoes

Oviposited and non-oviposited *Anopheles* mosquitoes were separated into head and thorax and abdomen then the genomic DNA (gDNA) of each mosquito was extracted using the Livak protocol with slight modification [22]. A cocktail and SINE PCR were used to identify sibling species belonging to *An. funestus* group and *An. gambiae* complex [23, 24].

### *Plasmodium* infection rate

gDNA from head and thoraxes and abdomens of F_0_ non-oviposited *An. funestus* s.s. and *An. gambiae* s.s. were used to detect *Plasmodium* species sporozoites and oocysts using TaqMan assay as previously described [25].

### Insecticide susceptibility assay

The susceptibility profile of *An. funestus* s.s. and *An. gambiae* s.s. to the four classes of insecticides was assessed using the WHO tubes bioassays [16]. Mosquitoes from the three [3] villages were tested for pyrethroids type I (permethrin 1x) and type II (alpha-cypermethrin 1x and deltamethrin 1x), the organochlorine (DDT 1x), carbamate (bendiocarb 1x and 5x), and organophosphates (malathion 1x and pirimiphos-methyl 1x). All the tests were performed at standard insectary conditions of 25 ± 2 °C temperature and 80 ± 10% relative humidity. Indeed, 20 to 25 F_1_ female *Anopheles* of 3 to 5 days old were put in holding tubes and observed for 1 h. After which, they were exposed to insecticides-impregnated papers for 1 h. Mosquitoes were transferred in holding tubes at the end of the exposure then knockdown was read, and tubes were supplemented by 10% sugar solution. Final mortality was read 24 h post-exposure and the results were interpreted according to the WHO guideline [16]. Two unexposed control tubes were used during the test to validate the experiment.

Based on the results obtained after exposure of mosquitoes at DC (i.e., 1x), an intensity test was carried out with 5x and 10x to assess the strength (intensity) of the resistance according to the WHO guidelines [16]. A synergist assay with piperonyl butoxide (PBO), an inhibitor of cytochrome P450 enzymes, was used to investigate the role of these enzymes in the ability of mosquitoes to survive exposure to insecticides.

### Insecticide-treated bed nets efficacy assays

Investigation of the bed nets efficacy was done following the WHO guidelines for Laboratory and Field-Testing of long-Lasting Insecticidal Nets [26]. The standard nets (DuraNet, PermaNet 2.0, Olyset and Interceptor), PBO-based nets (Olyset plus, PermaNet 3.0 side and PermaNet 3.0 top) and recent nets (Royal Guard and interceptor G2 made of alpha-cypermethrin + pyriproxyfen and alpha-cypermethrin + chlorphenapyr, respectively) were tested. An untreated net was used as the control during this experiment. Five replicates of 10 F_1_ mosquitoes of 2 to 5 days old females were placed in the plastic cone and exposed to the bed nets (treated) for 3 min. At the end of the exposure, mosquitoes were transferred in paper cups and supplemented with 10% sugar solution. Final mortality was recorded 24 h post-exposure and results were interpreted following WHO guidelines [26].

### Genotyping of resistance markers in *An. funestus* s.l. and *An. gambiae* s.l.

For *An. funestus* s.l., genotyping of *CYP6P9a*, *CYP6P9b*, *GSTe2*, 6.5 kb structural variants, GABA-RDL *A296S* and Ace-1 *N485I* were done using the abdomen of non-oviposited F_0_ mosquitoes. To link the molecular markers driving resistance with various phenotypes generated after exposure to pyrethroid type II (deltamethrin at 1x, 5x and 10x), the presence of *CYP6P9a* and *CYP6P9b* known to confer resistance to pyrethroids [3, 27], *GSTe2* known to confer resistance to DDT [28] and 6.5 kb structural variant known as an enhancer [29] was done on whole F_1_ mosquito alive and dead after exposure, as previously described.

For *An. gambiae* s.l., genotyping of *L1014S*-KdrE, *L1014F*-kdrW, *N1575Y* mutations linked to DDT and pyrethroids resistance, and *G119S*-ace-1 linked to organophosphate and carbamate resistance were done using the abdomen of non-oviposited mosquitoes by TaqMan assays [25, 30].

### Transcription profile of resistance genes in *An. funestus* s.s.

Total RNA of *An. funestus* mosquitoes were extracted from three [3] batches of 10 mosquitoes alive after exposure to deltamethrin 1x, 5x and 10x as well as for the control unexposed and FANG which is a highly susceptible laboratory colony originated from Angola [31]. The level of expression of *CYP6P9a*, *CYP6P9b*, *CYP6M7* and *GSTe2* was assessed by quantitative reverse transcription PCR (RT-qPCR) as previously described [32]. The relative expression was calculated according to the 2^−ΔΔCT^ method incorporating PCR efficiency after normalization with the housekeeping RSP7 ribosomal protein S7 (AGAP010592) and the actin 5C (AGAP000651) genes and compared between phenotypes as previously describe [33].

### Statistical analyses

The data was saved in an Excel file. The percentage of households with at least one LLIN was obtained by dividing the total number of nets seen and recorded by the households surveyed. The *Plasmodium* infection rate (PIR) was defined as the number of infected *Anopheles* mosquitoes out of the total number of mosquitoes tested, multiplied by 100.

The resistance profile of *Anopheles* vectors and bed nets efficacy was determined according to WHO guidelines [16, 26]. Indeed, the *Anopheles* vectors were susceptible if the mortality rate was ≥ 98%, probable resistant if the mortality rate was between 90 and 97% and resistant if the mortality rate was < 90%. The allelic frequency of each resistance marker was calculated based on the genotypic frequencies according to the formula below:

**f(R) = f(RR)** + **1/2f(RS)** with F(RR) the frequency of homozygote resistant mosquitoes and f(RS) the frequency of heterozygote mosquitoes.

**f(S) = 1** − **f(R)** with F(S) and f(R) as the frequencies of the susceptible and resistant allele respectively.

The association of each resistance marker with the phenotype of *Anopheles* mosquitoes exposed to deltamethrin 1x, 5x and 10x was done using Vassarstats to estimate the odds ratio (OR) based on a fisher exact probability test with a 2 × 2 contingency table and the differences observed were statistically significant if p < 0.05. Comparison of mean mortality rate between resistant mosquitoes at each concentration of insecticide was done by the Chi-square test and significance was set at 5%. Gene expression and fold-changes, relative to the susceptible laboratory colony *An. funestus* FANG was computed using the 2^−ΔΔCT^ method after standardisation with housekeeping genes (actin, 40s ribosomal protein S7; RPS7) [33]. Analyses were done using MS excel and R vs. 4.0.5 software and results were displayed as figures and tables.

## Results

### Mosquito sampling

Overall, 18 houses were surveyed in 3 areas of the Chikwawa district in Malawi. Seven (7), five (5) and six (6) houses in Medrum I, Medrum II and Npangueni respectively. All the houses were rectangular with thatched roof and with wall surfaces made of brick and mud (Additional file 1: Table S1, Table 1). The principal vector control tool in use in those villages was insecticide treated nets (ITNs), with bed net coverage of 71%, 80% and 83% for Medrum I, Medrum II and Npangueni respectively. Out of the houses screened, 74% (25/35) of the inhabitant from the 5 houses surveyed in Medrum I were protected by bed nets, 76% (16/21) and 88% (28/32) of them were protected in Medrum II and Npangueni respectively. Both standard pyrethroids only nets (PermaNet 2.0 and Royal Sentry) and PBO-based nets (PermaNet 3.0) were found in those villages distributed as part of the 2019 campaign. The proportion of PBO-based nets (6%) in the three villages was significantly lower compared to the proportion of standard nets (94%) (Additional file 1: Table S1).

### Species composition

The indoor collection with aspirator yielded 2350 *Anopheles* species mosquitoes out of which the predominant species belonged to the *An. funestus* group with 89.36% (2100/2350) followed by *An. gambiae* complex with 10.64% (250/2350). The oviposition rate was similar for both *An. funestus* s.l. and *An. gambiae* s.l. (51% vs. 48%, respectively).

### Molecular identification of field collected mosquitoes

**Fig. 2:**
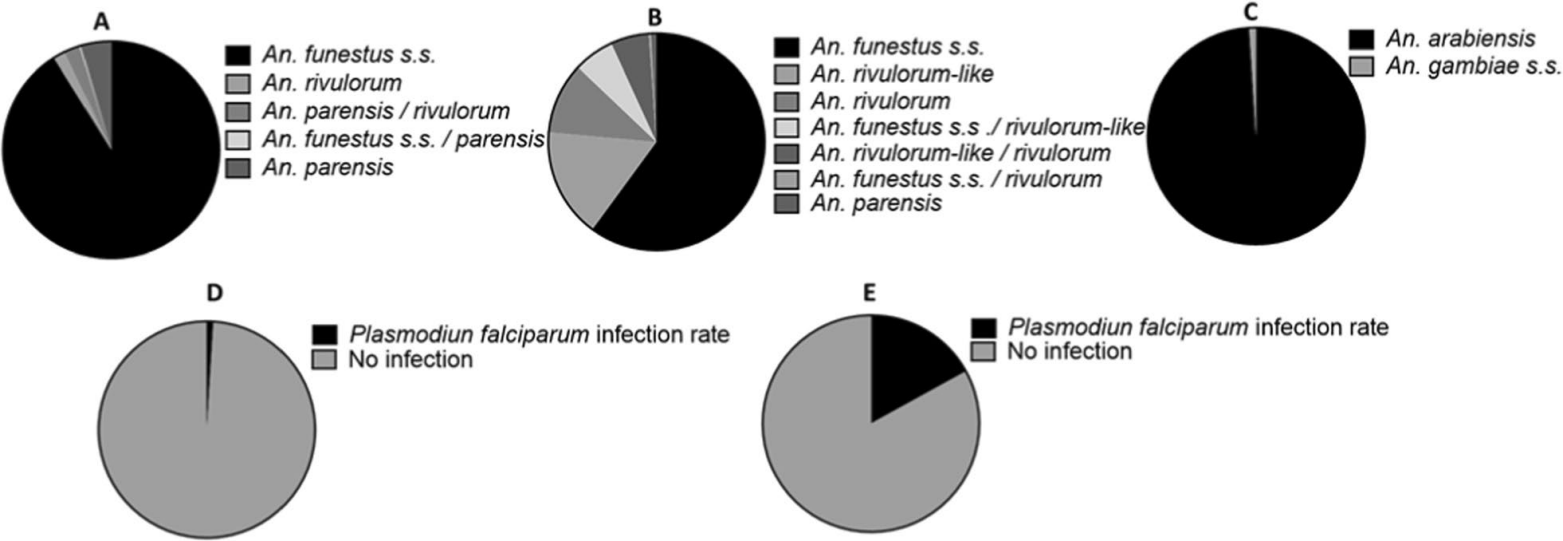
Species diversity and *Plasmodium* infection rate in Chikwawa. **A** Species composition within the *An. funestus* group in Chikwawa in June 2021. **B** Species composition within the *An. funestus* group in Chikwawa in January 2014. **C** Species composition within the *An. gambiae* complex in Chikwawa in June 2021.** D** Oocysts rate within the *An. funestus* s.s. **E** Sporozoites rate within the *An. funestus* s.s.

Molecular identification of a subset of 506 F_0_ oviposited *An. funestus* s.l. whole mosquitoes from the study area by cocktail PCR showed that 91.1% (461/506) were *An. funestus* sensu stricto, 4.54% (23/506) was *An. parensis*, 1.77% (9/506) was *An. rivulorum*, 2.33% (12/506) were hybrid *An. parensis*/*An. rivulorum* and 0.19% (1/506) was hybrid *An. funestus s.s.*/*An. parensis.* These results suggest that *An. funestus* s.s. is the principal species in Chikwawa (Fig. 2A). In 2014 a bigger number of species from *An. funestus* group were found in Chikwawa (Fig. 2B).

Out of a subset of 90 F_0_ non oviposited *An. gambiae* s.l. abdomens tested with SINE PCR for sibling species identification, 99% (89/90) were *An. arabiensis* and 1% (1/90) was *An. gambiae* s.s. revealing that *An. arabiensis* is the main vector in the *An. gambiae* complex in Chikwawa (Fig. 2C).

### *Plasmodium* infection rate

TaqMan assay was used to detect infection with *Plasmodium* in *An. funestus* s.s. and *An. arabiensis* using head and thoraxes (for sporozoite detection) and abdomens (for oocyst detection). Out of a subset of 98 F_0_ head and thorax of non-oviposited *An. funestus* s.s. tested, sporozoites were present in one *mosquito* (1.02%) belonging to *P. falciparum* (Fig. 2E). For the abdomens tested for the detection of oocysts, the infection rate was 17.30% (9/52) (Fig. 2D).

None of the 90 F_0_ non-oviposited *An. arabiensis* head and thoraxes nor abdomens tested for the detection of *Plasmodium* species sporozoites or oocysts were found to be infected.

### Insecticide susceptibility profile and intensity

#### Susceptibility profile of *An. funestus* s.s. from Chikwawa with discriminating concentration 1x (DC)

**Fig. 3:**
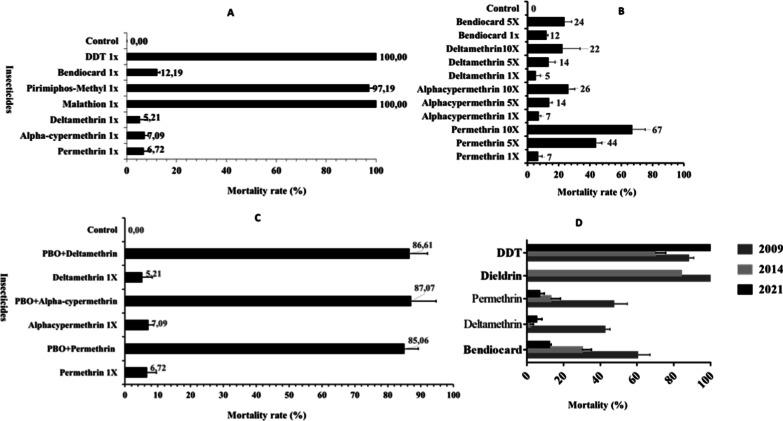
Insecticide susceptibility profile and intensity. **A** Susceptibility pattern of *An. funestus* s.s. mosquito populations from Chikwawa to DC of all the insecticides classes. **B** Resistance intensity pattern of *An. funestus* s.s. mosquito populations from Chikwawa to pyrethroids and carbamate. **C** Synergist assay displaying the effect of PBO on *An. funestus* s.s. population from Chikwawa. **D** Escalation of resistance from 2009 to 2021 in Chikwawa population

First generation (F_1_) adult *An. funestus* female collected in the three villages of Chikwawa demonstrated a high resistance to both pyrethroids type I (permethrin 1x) and type II (alpha-cypermethrin 1x and deltamethrin 1x) and to carbamate (Bendiocarb 1x). F_1_ females showed 6.72 ± 2.81%, 7.09 ± 1.62%, 5.21 ± 3.13% and 12.19 ± 1.10% mortality rate 24 h post-exposure to permethrin 1x, alpha-cypermethrin 1x, deltamethrin 1x, and bendiocarb 1x respectively. A near susceptibility was observed with organophosphate (pirimiphos-methyl 1x) with 97 ± 1.70% mortality rate at 24 h post-exposure. Full susceptibility was noted in this population with organochlorine (DDT) and organophosphate (Malathion) with 100% mortality rate (Fig. 3A).

#### Intensity assay with pyrethroids type I (permethrin), type II (alpha-cypermethrin and deltamethrin) and carbamate (bendiocarb 5x)

Due to the high resistance observed in *An. funestus* s.s. to pyrethroids and carbamate, intensity assay was done to assess the strength of resistance with 5x and 10x the DC of permethrin, alpha-cypermethrin, deltamethrin and bendiocarb. *An. funestus* s.s. from Chikwawa showed mortality rates of 43.8 ± 3.7%, 13.9 ± 2.1%, 13.5 ± 4.2% and 23.6 ± 4.6% to permethrin 5x, alpha-cypermethrin 5x, deltamethrin 5x and bendiocarb 5x, respectively meaning a moderate to high intensity of resistance in the locality (Fig. 3B). However, a significant increase in mortality was observed in *An. funestus* s.s. when moving from permethrin 1x to 5x and 10x (X² = 32.9, p < 0.0001) and from bendiocarb 1x to 5x and 10x (X² = 4.4, p = 0.03). The mortality rate 24 h post-exposure to 10 times DC of insecticides were 66.8 ± 8.5%, 26.1 ± 4.3%, 22.4 ± 11.3% for permethrin, alpha-cypermethrin and deltamethrin, respectively. This indicated extremely high intensity of resistance in the *An. funestus* population from Chikwawa. However, a significant increase in mortality rates was noticed in *An. funestus* s.s. when moving from permethrin 5x to 10x (X² = 9.9, p = 0.0016) and from alpha-cypermethrin 5x to 10x (X²= 4.1, p = 0.04) (Fig. 3B).

#### Synergist assays with PBO

Pre-exposure of *An. funestus* s.s. to PBO for 1 h then to permethrin 1x, alpha-cypermethrin 1x and deltamethrin 1x for another 1 h resulted in a significant recovery of susceptibility in these mosquitoes 24 h post-exposure with 85.06 ± 4.22% for PBO + permethrin 1x vs. 6.7 ± 2.8% for permethrin 1x (X² = 116.2, p < 0.0001), 87.1 ± 7.7% for PBO + alpha-cypermethrin 1x vs. 7.1 ± 1.6% for alpha-cypermethrin 1x (X² = 115.4, p < 0.0001) and 86.6 ± 5.5% for PBO + deltamethrin vs. 5.2 ± 3.1% for deltamethrin (X² = 117.5, p < 0.0001) (Fig. 3C). These results suggest that P450s enzymes are partially driving the high resistance observed in *An. funestus* s.s. mosquito population in Chikwawa.

#### Escalation of resistance from 2009 to 2021

Levels of the Chikwawa population in 2021 indicated an overall increase in resistance towards pyrethroids but not for DDT. For pyrethroids, the mortality rate for permethrin reduced from 47.2% in 2009 [21] to 13% in 2014 [20] and 6.7% in 2021 (Fig. 3D). For deltamethrin, the mortality rate reduced from 42.3% in 2009 to 1.75% in 2014 and 5.2% in 2021 (Fig. 3D). For Bendiocarb, the mortality rate reduced from 60% in 2009 to 30.1% in 2014 and 12.2% in 2021 suggesting an ongoing selection for this insecticide class (Fig. 3D). For the DDT, we rather noticed a recovery of the susceptibility in 2021 with a full susceptibility observed after resistance was noted in 2009 (87.8% mortality) and 69.91 in 2014 (Fig. 3D).

#### Insecticide-treated Bed Nets efficacy assays

**Fig. 4:**
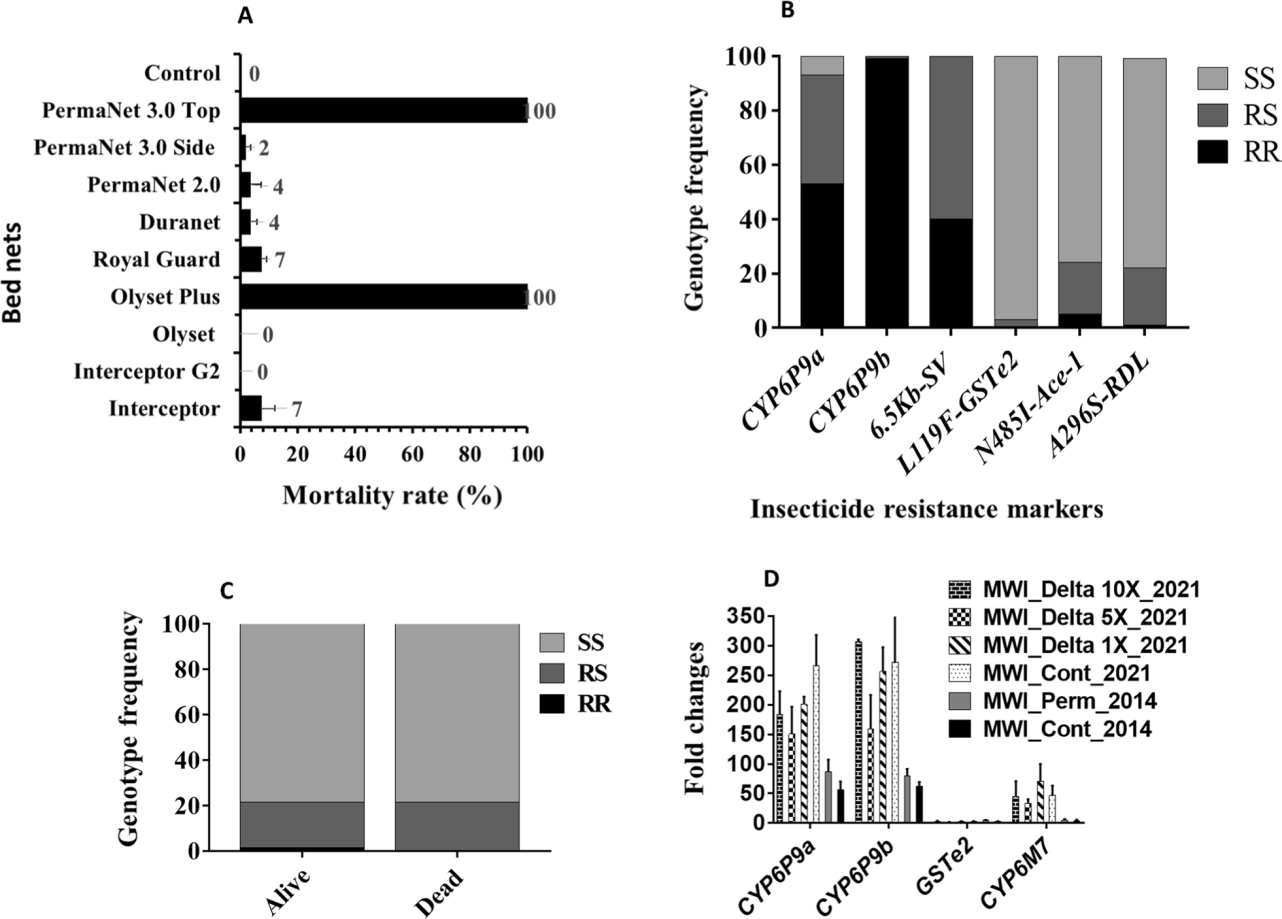
Cone assays with various nets against *An. funestus*, distribution of major markers and the association with resistance escalation. **A** Bioefficacy of net following 3-min exposure by cone assay against *An. funestus* s.s. **B** Distribution of major resistance markers in the *An. funestus* s.s. population from Chikwawa. **C** Detection of the three genotypes of Ace-1 *N485I* in *An. funestus* from Chikwawa after exposure to Bendiocarb. **D** Differential gene expression of the P450 genes cluster in *An. funestus* s.s. populations from Chikwawa

Cone assays were done to evaluate the influence of super-resistance on the efficacy of control intervention tools such as ITNs including Royal Guard and Interceptor G2. A very low efficacy of standard bed nets notably pyrethroid-only nets was observed against *An. funestus* from Chikwawa: 3.6 ± 3.4%, 0%, 3.7 ± 2.3%, 7.3 ± 4.75% mortality rates for PermaNet 2.0, Olyset, DuraNet and Interceptor respectively. Surprisingly, recent nets including Royal guard (made of alpha-cypermethrin + pyriproxyfen) and Interceptor G2 (made of alpha-cypermethrin + Chlorphenapyr) exhibited very low efficacy against *An. funestus* s.s. 0% and 7.3 ± 1.84% respectively (Fig. 4A). However, PBO-based net (PermaNet 3.0 top: 100.0 ± 0.0% and Olyset plus: 100.0 ± 0.0%) showed high efficacy against these vectors except for PermaNet 3.0 side (1.8 ± 1.8%). No significant difference in mortality rate was observed between PermaNet 2.0 (3.6 ± 3.4%) and PermaNet 3.0 side [(1.8 ± 1.8%) X² = 0.32, p = 0.57].

#### Genotyping of insecticide resistance markers in *An. funestus* s.s. and *An. gambiae* s.l.

Key resistance markers were genotyped in F_0_ mosquitoes to assess their frequencies and association with resistance phenotypes in Chikwawa. Out of 99 F_0_ field collected *An. funestus* s.s. genotyped for detection of *CYP6P9a*, *CYP6P9b*, *GSTe2*, 6.5 kb structural variant, Ace1-*N485I* and *A296S*-RDL, the frequency of the mutation was 66.7% (48 RR, 36RS and 5 SS) for *CYP6P9a*, 99.4% (89RR, 1RS and 0SS) for *CYP6P9b*, 69.9% (37RR, 56RS and 0SS) for *6.5 kb* structural variant, 1.7% (0RR, 3RS and 85SS) for *GSTe2* 14.8% (5RR, 19RS and 74SS) for Ace1-*N485I* and 11.7% (1RR, 21RS and 76SS) for *A296S*-RDL (Fig. 4B).

Genotyping of non-oviposited *An. arabiensis* for the detection of *L1014S*-kdr, *L1014F*-kdr *N1575Y*-Kdr and *G119S*-Ace1 reveals that none of these markers were present in these species in Chikwawa.

#### Correlation between the *CYP6P9a*, *CYP6P9b* and 6.5 kb SV markers and escalation of pyrethroid resistance

**Fig. 5:**
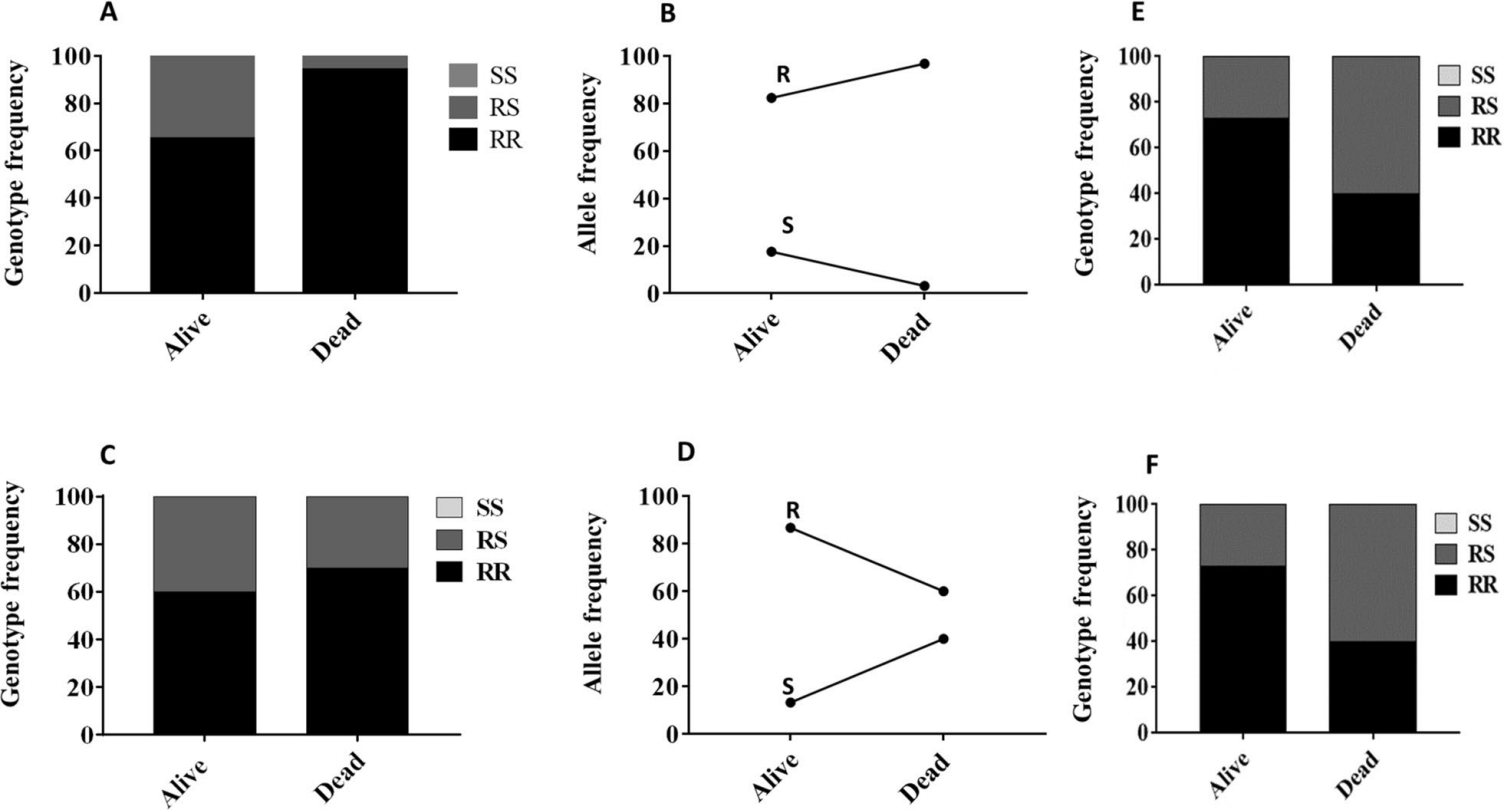
Correlation between the *CYP6P9a, CYP6P9b* and 6.5 kb *sv* markers and escalation of pyrethroid resistance. **A** Genotype distribution of *CYP6P9a* between alive and dead mosquitoes after 5x deltamethrin exposure; **B** allelic frequency of *CYP6P9a* between alive and dead mosquitoes after 5x deltamethrin exposure; **C** genotype distribution of *CYP6P9a* between alive and dead mosquitoes after 10x deltamethrin exposure; **D** allelic frequency of *CYP6P9a* between alive and dead mosquitoes after 10x deltamethrin exposure; **E** genotype distribution of 6.5 kb *sv* between alive and dead mosquitoes after 5x deltamethrin exposure; **F** genotype distribution of 6.5 kb *sv* between alive and dead mosquitoes after 10x deltamethrin exposure

The *CYP6P9b* was nearly fixed in this *An. funestus* population with only a single heterozygote detected out of 150 genotyped. Thus, this marker was not assessed for the correlation with resistance. For *CYP6P9a* using mosquitoes exposed to 5x deltamethrin, no homozygote susceptible was detected in both phenotypes. Consequently, a comparison was done only between homozygote resistant (RR) and heterozygotes (RS), revealing that dead mosquitoes after 5x deltamethrin exposure had significantly more chance of being homozygote resistant (Odds ratio (OR) = 0.12, Confidence interval: 0.049–0.3, P < 0.0001 (Fig. 5A). Overall, the resistant allele frequency was higher in dead (96.8%) than alive (82.4%) whereas the susceptible allele (S) was higher in alive (17.6%) than in dead (3.2%) (Fig. 5B). Similarly, with mosquitoes exposure to 10x deltamethrin, it was observed a higher proportion of RR in dead (70%) than in alive (60%) with an OR = 0.64, CI 0.36–1.15 although not significant (P = 0.18) (Fig. 5C, D).

Analysis of the genotyping patterns of the 6.5 kb *SV* rather indicated a significant association with resistance escalation with homozygote resistant (RR) more likely to survive exposure to 5x deltamethrin than heterozygotes OR = 3.1 (CI 1.49–6.3; P = 0.002) (Fig. 5E). Same pattern was observed at 10x with an OR = 4.1 (CI: 2.27–7.4, P < 0.0001) when comparing RR to RS (Fig. 5F).

**Fig. 6:**
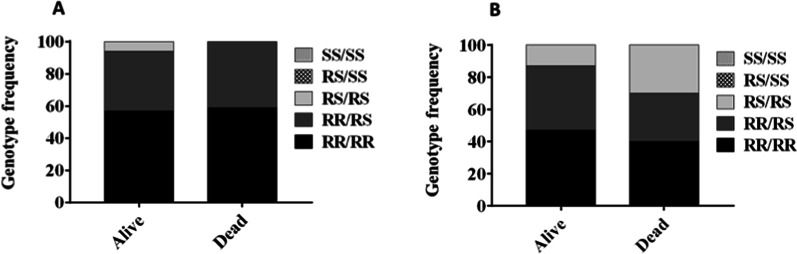
Correlation between the *CYP6P9a*, and 6.5 kb *SV* markers combined and escalation of pyrethroid resistance. **A** Genotype distribution *of CYP6P9a* combine to 6.5 kb *SV* between alive and dead mosquitoes after 5x deltamethrin exposure. **B** More double homozygote resistant are found in those surviving exposure to deltamethrin 10x

When combining both markers, no association was observed at 5x (Fig. 6A) whereas at 10x significantly more double homozygote resistant are found in those surviving exposure to deltamethrin with OR = 2.2 (CI 1.04–4.7; P = 0.02) (Fig. 6B). However, the number of dead genotyped at 5x and 10x was low.

#### Correlation between the Ace-1 *N485I* mutation and bendiocarb resistance

The assay, designed to genotype the Ace-1 *N485I* markers, unambiguously detected the three genotypes in 14 susceptible and 65 resistant mosquitoes against Bendiocarb 1x (Fig. 4C). The homozygote resistant RR to Ace-1 *N485I* markers were more able to survive exposure to bendiocarb 1x compared to homozygote susceptible SS, with odds ratio of 5.06 but not significantly (P = 0.29) (Table 1).

A significant association was observed between the Ace-1 *N485I* marker and bendiocarb 5x resistance, with an odds ratio of 2.89 (P < 0.006) when comparing heterozygote resistant (RS) to homozygote susceptible (SS) (Table 1).

**Table 1:** Association between the Ace-1 *N485I* and the ability of *Anopheles funestus* from Chikwawa to survive Bendiocarb 1x and Bendiocarb 5x

	OR	P value	CI
Mortality			
Bendiocarb 1x		*Ace-1 N485I*	
RR vs. SS	5.0637	P = 0.2976	0.239 to 107.177
RS vs. SS	0.9646	P = 0.9182	0.484 to 1.918
RR vs. RS	5.2439	P = 0.2942	0.237 to 115.952
R vs. S	1.1033	P = 0.8246	0.462 to 2.632
65 alive and 14 dead			
Bendiocarb 5x			
RR vs. SS	0.1070	P = 0.1325	0.0058 to 1.9674
RS vs. SS	2.8917	P = 0.0067	1.3424 to 6.2287
RR vs. RS	0.0380	P = 0.0313	0.0019 to 0.7453
R vs. S	1.3448	P = 0.5072	0.5603 to 3.2277

65 alive and 21 dead

#### Transcription profile of resistance genes in *An. funestus *s.s.

The expression level of duplicated genes *CYP6P9a* and *CYP6P9b*, *CYP6M7* and *GSTe2* known to confer resistance to pyrethroids and DDT in *An. funestus* reveals a major increase in expression of the three P450s compared to their levels in 2014 particularly for the duplicated *CYP6P9a/b* genes which correlate with the near fixation of the resistance alleles for these genes notably for *CYP6P9b* which exhibited the highest expression (Fig. 4D). For *CYP6P9a*, a 4.75-fold increased expression was observed for unexposed mosquitoes from 2014 (FC56.2) to 2021 (FC 266) (Fig. 4D). No significant difference was seen between 1x, 5x and 10x survivors as well as with control unexposed suggesting an overall high expression of this gene in the Chikwawa population with fold changes of 200.4 ± 13.2, 150.47 ± 46.2, 183.9 ± 39.2 and 266.6 ± 51.7 for deltamethrin 1x, 5x, 10x and control unexposed mosquitoes respectively. A similar pattern was observed for *CYP6P9b*, with a sharp temporal 4.4-fold increased expression for unexposed mosquitoes from 2014 (FC62) to 2021 (FC 272). Equally, no significant difference was seen between 1x, 5x and 10x survivors as well as with control unexposed with fold changes of 256.8 ± 40.7, 158.87 ± 6.10, 306.8 ± 3.9, 272.2 ± 135.2 for deltamethrin 1x, 5x, 10x and control unexposed, respectively. The *CYP6M7* also presented a marked temporal increased expression of 10.4-fold for unexposed mosquitoes from 2014 (FC4.4) to 2021 (FC46) (Fig. 4D) with similarly no significant difference of expression between all tested samples in 2021. No temporal change in expression was observed for *GSTe2* with lower expression < 3-fold compared to the susceptible FANG which also associates with the very low frequency of the resistant allele L119F-*GSTe2* in this population (< 3%). However, it should be noted that this allele was completely absent in 2014 [28].

## Discussion

Assessing the resistance intensity on the major malaria vectors as well as the associated mechanisms is important to anticipate the spread of such super-resistance regionally or across continent-wide. It also allows to better plan for the implementation of new intervention strategies in the field to progress towards malaria elimination. This study has extensively characterised the patterns of such resistance aggravation in the *An. funestus* population of southern Malawi revealing key findings about the extent, impact and molecular drivers of this super-resistance.

### A high species diversity in *An. funestus* group represents a challenge for malaria control

Here we revealed that the composition of the *An. funestus* group in Chikwawa in the southern Malawi is complex with several species identified. Molecular identification of a subset of 506 F_0_ oviposited *An. funestus* s.l. mosquitoes from Chikwawa revealed that although *An. funestus* was the predominant species (91.1%) other members of the group were present with also hybrids between these species. This complex vector composition was already revealed in 2014 in Chikwawa with four species and a large number of hybrids reported [20]. This high diversity in species composition is not specific to the southern of Malawi. Similar high diversity was also observed at Karonga in the northern part of Malawi [34]. This complex vector population within *An. funestus* group in Malawi highlights the need for accurate species identification for this species group all over the country. This will help the national control malaria program to have reliable entomological data for malaria control. The proportion of hybrids in this study and in previous studies in Chikwawa suggests an introgression phenomenon between the members of *An. funestus* group. This phenomenon of introgression taking place may lead to the exchange of genes between these species impacting the resistance profile or the susceptibility to *Plasmodium*. Further studies are needed to investigate the resistance profile and to establish the *Plasmodium* infection rate in the other species of the group besides *An. funestus* s.s. to see how this introgression may impact malaria transmission and control in this region.

### *Plasmodium* infection rate in Chikwawa

Overall, a low sporozoite infection rate was observed in this *An. funestus* population of Chikwawa (1.02%). This is similar to previous report in the same population back in 2014 [20] suggesting that the high resistance level observed here may not result to a spike in malaria transmission. However, a higher infection rate was observed when assessing the abdomens with an oocyst infection rate of 17.30% (9/52) suggesting that there is a significant circulation of *Plasmodium* in the population. The higher infection rate found in abdomen (17.30%) compared to head and thorax (1.02%) in this study further supports the significant barriers that the midgut plays in preventing oocyst migration to salivary gland [35]. Further investigations are needed to establish the role of other species such as *An. rivulorum* which has been reported as minor malaria vector in Tanzania [36] or Kenya [37] in malaria transmission in Chikwawa.

### An escalation of pyrethroid resistance and multiple resistance to other insecticide classes is worrying for control strategies in Malawi

Here we report a drastic increased level of insecticide resistance and the development of the intensity of the resistance over 10 years in *An. funestus* s.s. from Chikwawa. This increase is a concern for malaria control program since *An. funestus* s.s. is the predominant malaria vector in this African region and in Malawi. This resistance is higher to the main insecticides used for bed nets impregnation for malaria control such as pyrethroid. These *An. funestus* populations were resistant to permethrin and deltamethrin at all diagnostic concentrations of 1x, 5x, and 10x. This study is the first evidence of resistance escalation in *An. funestus* in Malawi using the 5x and 10x DC of pyrethroids. However, the high level of resistance to the diagnostic dose (1x) of pyrethroids in *An. funestus* is higher compared to the temporal increase in pyrethroid resistance observed in Chikwawa in 2009 and 2014 [11]. This resistance to permethrin deltamethrin and alphacypermethrin at all diagnostic concentrations of 1x, 5x, and 10x was also recently observed in *An. funestus* population from Uganda [8]. The susceptibility to Pirimiphos-methyl was observed in Chikwawa showing that organophosphates can be an alternative to pyrethroid resistance for IRS. Currently, IRS in Malawi uses three insecticides, Actellic 300CS, SumiShield, and Fludora Fusion in rotations.

The exposure to insecticide after exposure to PBO showed a high recovery of the mortality suggesting that P450 is playing a role in resistance observed. These results are in line with the high mortality observed against Olyset Plus and PermaNet 3.0 which are PBO based nets. This suggests that PBO nets can be considered as a solution for malaria control in this location and overall against *An. funestus* in southern Africa which tends to mainly exhibit a P450-based resistance mechanism [32, 38]. Nevertheless, the different trend observed in Mozambique with only a moderate mortality against PBO based nets [14] with P450 *CYP9P9a/b* resistance alleles that have become fixed points to the risk of also losing the efficacy of these PBO-based nets if resistance intensity continued unabated. In *An. funestus* from Uganda low mortality after exposure to permethrin + PBO and deltamethrin + PBO together with the loss in the efficacy of the new generation of PBO based nets particularly the Olyset plus was also observed [8]. Noticeably, the resistance to carbamates also significantly increased in this population despite this class not being used for vector control. This is in contrast to Uganda where resistance escalation was confined only to pyrethroids [8]. This could be an indication of a cross resistance between pyrethroids and carbamates in this Malawian *An. funestus* population. The causes of such high resistance could be associated with the scale-up of LLINs distribution across the country as bed nets across the country. The massive use of pyrethroids in agriculture in Chikwawa could be another factor in selecting for resistance in malaria vectors. We also noticed a recovery of the susceptibility to DDT from 69.9% in 2014 to 100% in 2021, supporting that resistance reversal is possible in the field once the source of selection disappears or is withdrawn. Consequently, a rotation-based resistance management strategy could be efficient in reducing level of resistance notably in the presence of fitness cost as shown in *An. funestus* for major resistance genes [39, 40].

### Increased frequencies and expression of P450 alleles partly explain the aggravation of pyrethroid resistance

The greater resistance to pyrethroids in this population is associated with increased expression of key cytochrome P450s previously shown to drive resistance, such as *CYP6P9a*, *CYP6P9b* and *CYP6M7* [32, 41]. This further supports previous observations that these genes are the main drivers of pyrethroid resistance in southern African *An. funestus.* However, there are differences in the allelic frequency of these markers with a complete fixation for *CYP6P9b*, whereas this is not the case with the other duplicated gene *CYP6P9a.* This supports a greater role of *CYP6P9b* in the resistance this location as shown with previous transcriptomic results with microarray [32] and RNAseq [38] that *CYP6P9b* is more over-expressed in Malawi which is the opposite in southern Mozambique [32]. Equally the 6.5 kb shown to be an enhancer of the expression of both *CYP6P9a/b* was not fixed although its significant correlation with the ability to survive exposure to 5x and 10x deltamethrin suggests that it is playing an important role in the observed escalation. The lack of correlation between expression of *CYP6P9a/b* and *CYP6M7* and survivorship to greater concentration of insecticide is similar to results for *CYP9K1* in Uganda whose expression although high was similar for mosquitoes surviving 1x, 5x or 10x [8] suggesting that other molecular factors are driving the ability of mosquitoes to survive exposure to greater concentrations of insecticides. A noticeable feature was the detection for the first time of the L119F-GSTe2 allele in a southern African population of *An. funestus* as this allele shown to confer pyrethroid/DDT resistance was previously confined to West and Central Africa and part of east Africa (Uganda) [41, 42]. It remains to establish if this is a de novo selection of this allele in Malawi or this is the result of gene flow which has been shown previously to be limited between southern Africa and other African populations of this species [43, 44]. It is an additional concern that an increase frequency of the L119F*-GSTe2* could worsen the resistance against pyrethroids in this population leading also to loss of efficacy of even PBO-based nets as shown in Cameroon where L119F-*GSTE2* was shown to reduce efficacy of PBO-based nets in experimental huts [45].

## Conclusions

The aggravation of the resistance against pyrethroids in Malawi represents a serious challenge for vector control interventions. The high mortality observed against PBO nets suggests that these nets are still a solution in the area but their performance should continually be monitored. However, the greater complexity of molecular drivers of this resistance escalation with new factors such as the new detection of the L119F-*GSTe2* resistance allele may further reduce the efficacy of existing control tools. This thus calls for the use of new tools to maximise the control of malaria vectors in the region. The overall, susceptibility to organophosphates opens the option for indoor residual spraying together with new insecticides such as neonicotinoids.

## Supplementary Information

**Additional file 1: Table S1.** Household indices and brand nets from the collection sites.

## Data Availability

All data generated or analysed during this study are included in this published article and its Additional files.

## Abbreviations

An: Anopheles
DC: Discriminating concentration
SV: Structural variant
OD: Odds ratio
FC: Fold change
PBO: Piperonyl butoxide
DDT: Dichlorodiphenyltrichloroethane
