# Supplementary material for: Marked aggravation of pyrethroid resistance in major malaria vectors in Malawi between 2014 and 2021 is partly linked with increased expression of P450 alleles
Source: BMC Infect Dis. 2022 Jul 30;22:660. doi: 10.1186/s12879-022-07596-9 (PMC9338535; doi:10.1186/s12879-022-07596-9)
Supplement: Supplementary file 1 — Additional file 1: Table S1. Household indices and brand nets from the collection sites. [file 12879_2022_7596_MOESM1_ESM.docx]

**Additional file 1**

**Table S1:** Household indices and brand nets from the collection sites.

| Household Indices | Medrum I | Medrum II | Npangeni |
| --- | --- | --- | --- |
| Number of houses | 7 | 5 | 6 |
| Houses with net | 5 | 4 | 5 |
| Bed net coverage | 71% | 80% | 83% |
| Number of inhabitants protected | 35 | 21 | 32 |
| Protection rate | 74% | 76% | 88% |
| Brands of nets used | **Medrum I** | **Medrum II** | **Npangeni** |
| PermaNet 2.0 | 5 (100%) | 5 (83%) | 5 (83%) |
| PermaNet 3.0 | 0 (0%) | 0 (0%) | 1 (17%) |
| Royal Sentry | 0 (0%) | 1 (17%) | 0 (0%) |
| Total | 5 (100%) | 6(100%) | 6 (100%) |
